# Editorial: The innate and adaptive immune system in human urinary system

**DOI:** 10.3389/fimmu.2023.1294869

**Published:** 2023-10-09

**Authors:** Payam Behzadi, Cheorl-Ho Kim, Edyta Agnieszka Pawlak, Abdelazeem Algammal

**Affiliations:** ^1^ Department of Microbiology, Shahr-e-Qods Branch, Islamic Azad University, Tehran, Iran; ^2^ Molecular and Cellular Glycobiology, Department of Biological Sciences, College of Science, Sungkyunkwan University, Suwon, Republic of Korea; ^3^ Head of Laboratory of Immunopathology, Department of Experimental Therapy, Hirszfeld Institute of Immunology & Experimental Therapy, Polish Academy of Sciences, Wroclaw, Poland; ^4^ Department of Bacteriology, Immunology, and Mycology, Faculty of Veterinary Medicine, Suez Canal University, Ismailia, Egypt

**Keywords:** innate immunity, adaptive immunity, human urinary system, nervous system, interleukins, toll-like receptors, cytokines

The urinary, immune, and nervous systems communicate with each other to create an effective network in a healthy human body. The kidneys and the bladder are known as the main parts of the urinary system and are joined together via ureters (1–3). The urinary system maintains homeostasis through the innate and adaptive immune cells (such as macrophages (MΦs)). These immune cells express a wide range of immune biomolecules including interleukins (ILs) and pattern-recognition receptors (PRRs) e.g., toll-like receptors (TLRs). Due to this knowledge, microglia as specialized MΦs of the central nervous system (CNS) are effective phagocytotic cells that produce different types of PRRs such as TLRs. The functions of microglia are regulated by pro-inflammatory and anti-inflammatory cytokine receptors (4–6). Hence, both the immune and the nervous systems have a pivotal role in the maintenance of homeostasis in the human urinary system.

Because of the importance of this topic, the editors decided to run the present impactful Research Topic in the prestigious journal of Frontiers in Immunology. Our purpose was to collect a treasure trove of strong and useful research studies. Fortunately, we succeeded in our attempt to collect six thematic publications from 54 international authors.

In a cross-sectional study, Qin et al. investigate the systematic immune-inflammation index (SII) as a novel inflammatory marker and its association with albuminuria. In this regard, they obtain the related data including the SII and urinary albumin-to-creatinine ration (ACR) pertaining to 36,463 adults (females=50.96% males=49.04%) during 13 years between 2005 and 2018 from National Health and Nutrition Examination Survey (NHANES). A wide range of covariates e.g., race, gender, body mass index (BMI), age, diabetes, behavioral condition comprising smoking, etc. are included in this study. Their findings show a positive relationship between SII and enhancement of urinary albumin excretion among US adults in this survey.

In another investigation, Wang et al. study the correlation between the profile of urinary complements in patients with IgA nephropathy (IgAN) and the associated clinical and pathological characteristics. This survey was performed over a period of three years (2019-2022) regarding 213 urine samples taken from 213 individuals (68 individuals were healthy controls, while the remaining persons were patients with IgAN (n=95) and membranous nephropathy (MN) (n=68)) at Sichuan Provincial People’s Hospital, China. Wang et al. focus on urinary complement proteins. This team demonstrate the presence of 44 complement proteins in patients with IgAN. They also show that an unusual increase of complement proteins in patients with IgAN is in association with renal histological damage, decreased renal function, and proteinuria. Hence, Wang et al. suggest that the urinary complement profile could be recognized as a potential biomarker panel to monitor and treat patients with IgAN.

Park et al. demonstrate the inhibitory effects of lomerizine on lipopolysaccharide (LPS)-mediated neuroinflammation and tau hyperphosphorylation through modulating NLRP3, DYRK1A, and GSK3α/ßLPS in both *in vitro* and *in vivo* conditions. Lomerizine acts as an L- and T-type voltage-gated calcium channel antagonist. Microglia as a part of innate immune cells are activated in CNS through the microbial components e.g., LPS, or other stimuli including neural damages, injuries, etc. Their findings show that lomerizine can be recognized as a potential therapeutic alternative in patients with neuroinflammation- or tauopathy-related diseases.

Eggers et al. present the effect of a German-licensed vaccine, Stro Vac^®^ (Strathmann, Hamburg), which is used against recurrent urinary tract infections (rUTIs). This vaccine is composed of an adjuvant (aluminum phosphate) and ten heat-inactivated bacterial pathogens including six different strains of *Escherichia coli* and one strain of four different species such as *Enterococcus faecalis*, *Klebsiella pneumoniae*, *Morganella morganii*, and *Proteus mirabilis*. They show that this vaccine is able to activate both the innate and adaptive immune systems against the etiological agents of rUTIs.

Seo et al. investigate the potential role of urinary exosomal microRNA biomarkers as an innovative molecular diagnostic approach for acute rejection (AR) in kidney transplant recipients (KTRs). This method is validated through the determination of urinary exosomal microRNA signatures as novel and non-invasive biomarkers. In this regard, they study the urinary exosomal microRNA profiles pertaining to KTRs. The development and validation of the urinary exosomal microRNA profiles in KTRs is achieved by studying the published literature, recruitment of NanoString-based transcriptomics, and public databases. This team show that the urinary exosomal microRNA signatures could be recognized as suitable potential biomarkers to detect AR in KTRs; however, further investigations are needed.

The final article, written by Mazzarino et al., suggests two therapeutic methods regarding the reduction of chronic vascular inflammation (CVI) and lower cardiovascular (CV) pathology in patients with chronic kidney disease (CKD)-associated DAMPs. In this regard, the blood samples were taken from patients with CKD (stage 5). Mazzarino et al. use paquinimod to prevent calprotectin-TLR4/CD14 interaction in a single DAMP-targeting strategy, while they recruit soluble TLR2 (sTLR2) to prevent TLR2-DAMP interaction and CD14-dependent TLRs activation, and inhibit CD14 co-receptor activities in their second strategy named the multi-TLR blocking strategy. Simultaneously, the sTLR2 supports the suboptimal CD14-independent TLR activation. All in all, the former strategy decreases the CKD-associated vascular inflammation via inhibiting a single DAMP’s activity, while the latter strategy reduces the multiple DAMPs’ activities to decrease the CKD-associated vascular inflammation.

Replacement of the current antibiotic therapy with effective preventive procedures such as the consumption of vaccines, agonists, and antagonists may minimize the global concern regarding the classic pharmaceutical therapies and the related threats including the rise of antimicrobial resistance (AMR) feature, worldwide. Hence, this occurrence may be a promising alternative (7–10). Indeed, “prevention is better than cure”.

## Author contributions

PB: Writing – original draft, Writing – review & editing. CK: Writing – original draft, Writing – review & editing. EP: Writing – original draft, Writing – review & editing. AA: Writing – original draft, Writing – review & editing.
